# Purification, crystallization and preliminary X-ray diffraction analysis of the kinase domain of human tousled-like kinase 2

**DOI:** 10.1107/S2053230X14002581

**Published:** 2014-02-19

**Authors:** Ana M. Garrote, Pilar Redondo, Guillermo Montoya, Inés G. Muñoz

**Affiliations:** a Macromolecular Crystallography Group, Structural Biology and Biocomputing Programme, Spanish National Cancer Research Centre (CNIO), Melchor Fernández Almagro 3, 28029 Madrid, Spain; b Structural Biology Group, Novo Nordisk Foundation Center for Protein Research, Faculty of Health and Medical Sciences, University of Copenhagen, Blegdamsvej 3B, 2200 Copenhagen, Denmark

**Keywords:** human tousled-like kinase 2

## Abstract

Tousled-like kinases (TLKs) are an evolutionarily conserved family of serine/threonine protein kinases involved in chromatin dynamics, including DNA replication and repair, transcription and chromosome segregation. The two members of the family reported in humans, namely TLK1 and TLK2, localize to the cell nucleus and are capable of forming homo- or hetero-oligomers by themselves. To characterize the role of TLK2, its C-terminal kinase domain was cloned and overexpressed in *Escherichia coli* followed by purification to homogeneity. Crystallization experiments in the presence of ATP-γ-S yielded crystals suitable for X-ray diffraction analysis belonging to two different space groups: tetragonal *I*4_1_22 and cubic *P*2_1_3. The latter produced the best diffracting crystal (3.4 Å resolution using synchrotron radiation), with unit-cell parameters *a* = *b* = *c* = 126.05 Å, α = β = γ = 90°. The asymmetric unit contained one protein molecule, with a Matthews coefficient of 4.59 Å^3^ Da^−1^ and a solvent content of 73.23%.

## Abstract 2

The C-terminal kinase domain of TLK2 (a human tousled-like kinase) has been cloned and overexpressed in *Escherichia coli* followed by purification to homogeneity. Crystallization experiments in the presence of ATP-γ-S yielded crystals suitable for X-ray diffraction analysis belonging to two different space groups: tetragonal *I*4_1_22 and cubic *P*2_1_3.

## 1. Introduction   

The tousled-like kinases (TLKs) are a family of conserved serine/threonine protein kinases that are found in both plants and animals. They were named after the first member of the family to be described, tousled (TSL) protein, which is encoded by the *Arabidopsis thaliana* TSL gene (Roe, Nemhauser *et al.*, 1997 ▶; Roe *et al.*, 1993 ▶). Mutations in this gene result in abnormal flower development, affecting leaf morphology, flowering time and organogenesis (Roe, Nemhauser *et al.*, 1997 ▶; Roe *et al.*, 1993 ▶; Moshkin *et al.*, 2009 ▶). The human TLKs, namely TLK1 and TLK2, localize to the cell nucleus and interact with themselves, forming homo- or hetero-oligomers, as proven by yeast two-hybrid experiments (Roe, Durfee *et al.*, 1997 ▶; Silljé *et al.*, 1999 ▶). They share 84% similarity with each other and almost 50% similarity with *Arabidopsis* TSL (Takahata *et al.*, 2009 ▶). Both TLKs have their catalytic domains placed at the C-terminus, where the ATP-binding domain displays a G*X*G*XX*S motif instead of the canonical consensus sequence G*X*G*XX*G (Hanks *et al.*, 1988 ▶). In the N-terminal domain, conserved features include three potential nuclear localization sequences and three putative coiled-coil regions (Silljé *et al.*, 1999 ▶). 

The TLKs are regulated by cell-cycle-dependent phosphorylation and their activity is strongly linked to DNA replication, with maximal activity during the S phase. Also, they exhibit sensitivity to DNA-damaging agents and inhibitors of DNA replication (Takahata *et al.*, 2009 ▶). TLKs are involved in chromatin assembly through the binding and phosphorylation of the human chromatin assembly factors Asf1a and Asf1b (Silljé & Nigg, 2001 ▶), and have been associated with numerous replicative and transcriptional processes, including chromosome condensation and segregation (Sunavala-Dossabhoy *et al.*, 2003 ▶; Hashimoto *et al.*, 2008 ▶; Yap *et al.*, 2011 ▶), gene silencing (Wang *et al.*, 2006 ▶) and DNA repair (Li *et al.*, 2001 ▶; Canfield *et al.*, 2009 ▶); the latter is further supported by their role as targets of ATM and Chk1, two kinases that are involved in the DNA-damage checkpoint upon irradiation (Krause *et al.*, 2003 ▶). Recent studies have demonstrated their direct implication in the development of different types of cancer such as neuroblastoma and pancreatic and prostate cancers (Heidenblad *et al.*, 2005 ▶; Wang *et al.*, 2006 ▶; Ronald *et al.*, 2011 ▶), being key regulators of Kaposi’s sarcoma-associated herpesvirus and Epstein–Barr virus reactivation, since their expression is required for the maintenance of viral latency (Dillon *et al.*, 2013 ▶). This suggests the potential use of TLKs as biomarkers for diagnosis and prognosis, without neglecting the possibility of their use as drug targets affecting not only pathogenesis but also the response of the patient to chemotherapy and/or radiotherapy. In this study, we report the purification, crystallization, diffraction data collection and preliminary crystallographic analysis of the C-terminal kinase domain of TLK2.

## 2. Materials and methods   

### 2.1. Cloning and expression   

TLK2 is composed of 772 amino acids. The kinase domain (TLK2_KD; amino acids 451–752) was amplified by PCR using the cDNA of TLK2 as a template (GenBank accession ID gi:6063019). The PCR product was inserted into multiple cloning site 1 (MCS1) of pETDuet-1 vector (Novagen) in frame with an N-terminal hexahistidine, thus adding an MGSSHHHHHH purification tag. This vector includes the lambda protein phosphatase ORF at the MCS2, expression of which permitted the dephosphorylation of TLK2_KD to occur during its expression. The resulting construct was used to transform chemically competent *Escherichia coli* Rosetta pLysS cells grown in Luria–Bertani (LB) broth supplemented with 35 µg ml^−1^ chloramphenicol and 100 µg ml^−1^ ampicillin. Cells were cultured at 310 K and 200 rev min^−1^ for 3 h after induction with 0.3 m*M* isopropyl β-d-1-thiogalactopyranoside (IPTG) when the OD_600_ was between 0.6 and 0.8. Cells were then harvested by centrifugation at 4000 rev min^−1^ for 20 min at 277 K and cooled by immersion in liquid nitrogen until further use.

### 2.2. Purification   

**Figure 1:**
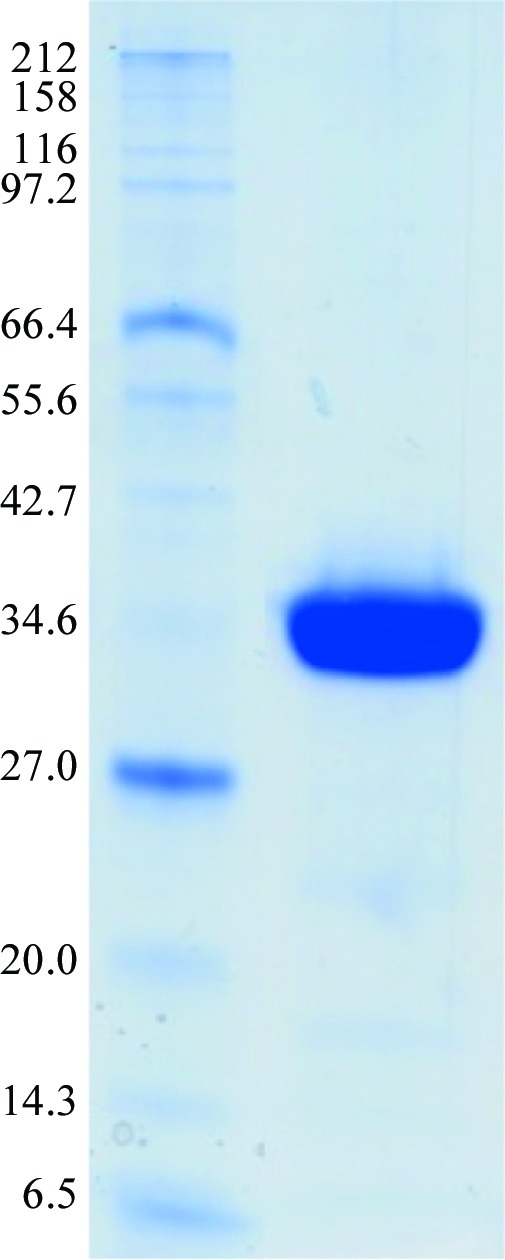
12% SDS–PAGE of purified TLK2_KD used for crystallization trials. Molecular-weight markers are labelled in kDa.

To purify TLK2_KD, the cell pellet was resuspended in 1:10(*w*:*v*) buffer *A* (50 m*M* Tris pH 8.8, 500 m*M* NaCl, 0.3 m*M* TCEP, 5% glycerol), including protease inhibitors (complete EDTA-free tablets, Roche). The resuspended cells were lysed by sonication (Sonic Dismembrator 500, Fisher Scientific) while cooling on ice for 15 min (2 s on/1 s off). The insoluble cell lysate was removed by centrifugation at 34 000*g* for 50 min at 277 K. We have estimated that approximately 80% of the protein is present in the soluble fraction. The supernatant was loaded onto a pre-equilibrated 5 ml Ni^2+^-loaded HisTrap Chelating HP column (GE Healthcare). The bound protein was eluted over 30 column volumes using a linear imidazole gradient to a final concentration of 100% buffer *B* (20 m*M* Tris pH 8.8, 25 m*M* NaCl, 0.3 m*M* TCEP, 5% glycerol, 500 m*M* imidazole). The elution peak of the kinase domain occurs at 12.6% buffer *B*, when it reaches a salt content of 440 m*M*. After pooling the protein fractions, the protein was loaded onto a HiTrap Q HP column (GE Healthcare) pre-equilibrated in buffer *C* (20 m*M* Tris pH 8.8, 25 m*M* NaCl, 0.2 m*M* TCEP). The high salt concentration in the loading sample avoids the binding of TLK2_KD to the column. The loading process was repeated several times to maximize the amount of protein contaminants bound to the column. In our experience, when the protein sample was dialyzed before loading against a buffer with lower salt concentration (50 m*M* NaCl), approximately 30% of the TLK2_KD was bound to the ion-exchange column along with other contaminants, thus considerably decreasing the final yield. The TLK2_KD fractions were concentrated to a final volume of 5 ml by centrifugation (3000 rev min^−1^ at 277 K) using an Amicon Ultra concentrator (Millipore) with a 10 kDa cutoff filter. The protein was then further purified and buffer-exchanged into buffer *D* (20 m*M* HEPES pH 7.5, 50 m*M* NaCl, 0.2 m*M* TCEP) by size-exclusion chromatography on a HiLoad 16/60 Superdex 75 column (GE Healthcare). Fractions corresponding to the purified protein were collected and concentrated to 12.3 mg ml^−1^ prior to crystallization. The homogeneity of the protein was determined by Coomassie Blue staining of a 12% SDS–PAGE gel (Fig. 1 ▶). The identity of the protein was confirmed by in-gel trypsin digestion (Sigma) and mass-spectrometric analysis (Proteomic Unit, CNIO, Madrid). The molecular mass of the protein was determined to be 36 695.8 Da by electrospray ionization mass spectrometry (ESI–MS), which matches the sequence-predicted mass of 36 696 Da and does not include the initial methionine (Frottin *et al.*, 2006 ▶).

### 2.3. Crystallization   

**Figure 2:**
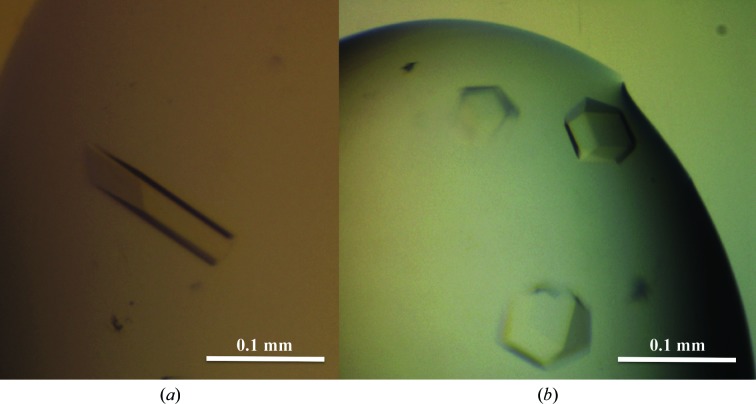
Crystals of TLK2_KD corresponding to space groups *I*4_1_22 (*a*) and *P*2_1_3 (*b*).

The crystallization trials were set up in 96-well sitting-drop plates (Innovadine) using a Cartesian MicroSys robot. The nanodrops were 0.2 µl in size, consisting of 0.1 µl reservoir solution plus 0.1 µl protein solution at 10 mg ml^−1^, and were equilibrated over 60 µl reservoir solution. The initial screening conditions were from the commercially available kits Crystal Screen, Crystal Screen 2 and Natrix (Hampton Research), Wizard I and II (Emerald BioSystems), JBScreen Kinase 1–4 and JBScreen Classic 1–10 (Jena), and The JCSG+ and Protein Complex Suites (Qiagen). For each screen the protein was tested either alone or in the presence of 2 m*M* ATP-γ-S. Duplicated crystal trays were stored at 277 and 291 K. These trials yielded crystals at 277 K under two conditions, Natrix condition No. 1 (crystal type *A*; 20 m*M* HEPES pH 7, 2 *M* Li_2_SO_4_, 10 m*M* MgCl_2_) and Wizard II condition No. 37 (crystal type *B*; 1 *M* sodium/potassium tartrate, 0.1 *M* Tris pH 7, 200 m*M* Li_2_SO_4_), and only when the protein was mixed with ATP-γ-S. Initial crystals were saved for seeding. Optimization of the crystallization conditions was performed by scaling up the initial hits using 24-well hanging-drop Linbro plates (Hampton Research). The crystals were reproducible using larger drops by mixing 1 µl reservoir solution and 1 µl protein solution at 5 mg ml^−1^, and in both cases they reached larger sizes when the reservoir solution was taken directly from the original Natrix and Wizard II kits (Fig. 2 ▶).

### 2.4. Data collection   

To prepare the crystals for data collection, they were cryoprotected by a 5 s soak in solutions consisting of 20 m*M* HEPES pH 7, 1 *M* Li_2_SO_4_, 10 m*M* MgCl_2_, 20% glycerol or 1 *M* sodium/potassium tartrate, 0.1 *M* Tris pH 7, 200 m*M* Li_2_SO_4_, 20% glycerol. They were then immediately vitrified in liquid nitrogen. Diffraction data were collected at 100 K on beamline XS06A at the Swiss Light Source (Villigen, Switzerland). 300 images of 0.5° oscillation per frame were collected from crystal type *A*, with a 620 mm crystal-to-detector distance and an exposure time of 0.5 s per image. 600 images of 0.2° oscillation per frame were collected from crystal type *B*, with a 500 mm crystal-to-detector distance and an exposure time of 0.2 s per image. Data were recorded for both crystals on a Pilatus detector; they were processed with *XDS* (Kabsch, 2010 ▶) and integrated and scaled using the *CCP*4 program suite (Winn *et al.*, 2011 ▶). Data-collection statistics are summarized in Table 1 ▶.

## 3. Results   

**Figure 3:**
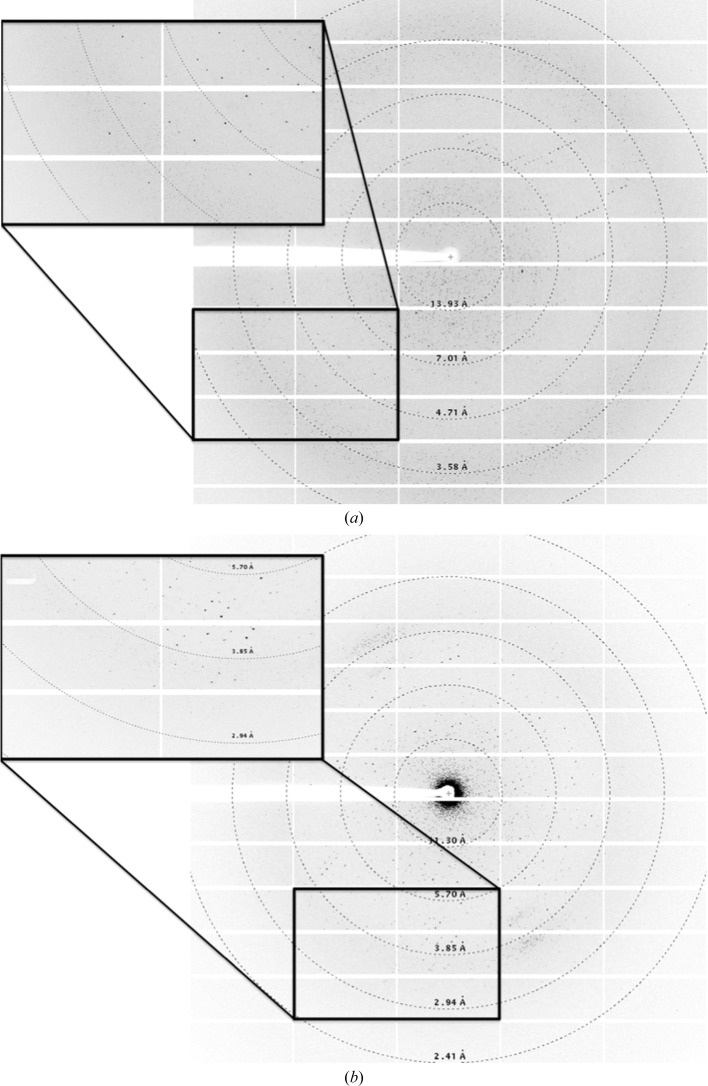
X-ray diffraction pattern images corresponding to the space groups *I*4_1_22 (*a*) and *P*2_1_3 (*b*).

The kinase domain of TLK2 (TLK2_KD) has been expressed and purified to homogeneity (Fig. 2 ▶). The yield of the pure protein was 1.7 mg per litre of culture. Purified TLK2_KD concentrated to 5 mg ml^−1^ produced crystals at 277 K under two different conditions, resulting in crystals belonging to two different space groups. The crystals grown using Natrix condition No. 1 diffracted to 3.7 Å resolution and belonged to space group *I*4_1_22, with unit-cell parameters *a* = *b* = 88.36, *c* = 342.87 Å, α = β = γ = 90°, and the crystals grown under Wizard II condition No. 37 diffracted to 3.4 Å resolution and belonged to space group *P*2_1_3, with unit-cell parameters *a* = *b* = *c* = 126.05 Å, α = β = γ = 90° (Fig. 3 ▶). Cell-content calculations (Matthews, 1968 ▶) predict that each asymmetric unit contains one molecule of TLK2_KD (*V*_M_ = 4.60 Å^3^ Da^−1^ for crystal type *A* and *V*_M_ = 4.59 Å^3^ Da^−1^ for crystal type *B*), with approximately 73% solvent in both cases, values that are consistent with the fragility of the crystals. Unfortunately, dehydration trials did not help to improve either their robustness or their resolution. Structure solution was obtained by molecular replacement using *Phaser* (McCoy *et al.*, 2007 ▶) with a search model generated by *I-­TASSER* (Roy *et al.*, 2010 ▶) that was mainly based on the structure of Aurora A kinase (PDB entry 3h10; Aliagas-Martin *et al.*, 2009 ▶), to which TLK2_KD is predicted to have a similar secondary structure (46% sequence homology). Refinement and model building are ongoing.

## Figures and Tables

**Table 1:** Data-collection statistics for the two TLK2_KD crystal forms Values in parentheses are for the outermost shell.

	Crystal form *A*	Crystal form *B*
Space group	*I*4_1_22	*P*2_1_3
Unit-cell parameters (Å, °)	*a* = *b* = 88.36, *c* = 342.87, α = β = γ = 90	*a* = *b* = *c* = 126.05, α = β = γ = 90
Data collection		
Temperature (K)	100	100
Wavelength (Å)	0.99987	1.00002
Beamline	XS06A, SLS	XS06A, SLS
Resolution (Å)	85.71–3.70 (3.90–3.70)	89.13–3.40 (3.58–3.40)
Total reflections	82242 (12186)	125158 (17818)
Unique reflections	7737 (1088)	9456 (1354)
Mulitplicity	10.6 (11.2)	13.2 (13.2)
Completeness (%)	100.0 (100.0)	100.0 (100.0)
Mean *I*/σ(*I*)	19.1 (3.5)	13.5 (3.6)
*R*_merge_ †	0.065 (0.79)	0.133 (0.71)

† *R*_merge_ is defined according to *XDS* (Kabsch, 2010 ▶).
